# From interaction to identification: a mixed methods study on the formation mechanism of group identity among Ultimate Frisbee club members

**DOI:** 10.3389/fpsyg.2026.1878237

**Published:** 2026-09-28

**Authors:** Ruyin Chen, Fangjin Chang, Kaijuan Xu

**Affiliations:** School of Economics and Management, Shanghai University of Sport, Shanghai, China

**Keywords:** group identity, interaction ritual chain theory, mixed methods, social exchange theory, Ultimate Frisbee

## Abstract

**Background:**

Lifestyle sports like Ultimate Frisbee have emerged as important settings for social connection amid urban social atomization. Nevertheless, the voluntary and non-contractual nature of these clubs means they continue to face persistent challenges in sustaining membership. Although prior research has explored group identity in various sporting contexts, the mechanisms through which ritualized interaction and social exchange jointly contribute to members' identification remain insufficiently understood.

**Objective:**

This study investigates the formation mechanism of group identity among Ultimate Frisbee club members by integrating Interaction Ritual Chain theory and Social Exchange theory, and empirically tests whether emotional energy and relational benefits serve as parallel mediating pathways.

**Methods:**

A sequential exploratory mixed-methods design was employed. Study 1 adopted a grounded theory approach, using semi-structured interviews with 22 club members across multiple Chinese cities to inductively identify core antecedents and construct a theoretical model. Study 2 employed a cross-sectional survey design, with structural equation modeling applied to a sample of 332 Ultimate Frisbee club members to quantitatively test the hypothesized pathways.

**Results:**

The grounded theory analysis indicated a dual-pathway mechanism encompassing an affective pathway (interaction rituals → emotional energy → group identity) and a rational pathway (interaction rituals → relational benefits → group identity). The SEM results indicated that both emotional energy and relational benefits significantly mediated the relationship between interaction rituals and group identity. The mediating effect of relational benefits—encompassing trust and social acceptance—was larger (33.58% of total effect) than that of emotional energy (14.97%).

**Conclusion:**

These findings integrate Interaction Ritual Chain theory and Social Exchange theory into a unified framework, suggesting that rational evaluations of social reciprocity were more strongly associated with identity formation than transient emotional experiences in non-contractual sports clubs. The results point to the central role of relational calculus in voluntary organizations where formal incentives are absent. From a practical standpoint, club managers may consider prioritizing relational rewards and the development of ritualized interaction settings to enhance long-term member retention.

## 1 Introduction

The acceleration of urbanization and the proliferation of digital technology have reshaped the structure of contemporary social life in China. Concurrent with the gradual weakening of traditional geographical and kinship ties, urban populations commonly face a dual dilemma: social atomization and a deficit of psychological belonging (Zhang, 2022; Wang et al., 2022). Within this context, the “neo tribes” described by Maffesoli (1996) have emerged. Individuals seek alternative community belonging through shared interests and emotional experiences. Among these emerging communities, Ultimate Frisbee clubs have grown considerably in recent years. These clubs function not only as venues for physical exercise but also as vital spaces for urbanites seeking social connection and psychological support (Gao et al., 2024; Li, 2022).

Ultimate Frisbee is a team sport characterized by unique features such as player self-officiating, mixed-gender rosters, and the “Spirit of the Game.” Despite the explosive growth and increasing institutional support for Ultimate Frisbee in recent years, clubs commonly confront persistent organizational challenges: high member turnover rates and low retention rates, and many clubs struggle to maintain a stable participant base. Member enthusiasm often wanes rapidly following the fading of initial novelty (Huang, 2024). Unlike professional sports organizations, Ultimate Frisbee clubs lack formal contractual constraints, financial remuneration, or institutional authority to bind members. Understanding the mechanisms underlying continued participation and psychological attachment is therefore of both theoretical and practical importance.

This study employs a two-stage mixed-methods approach to investigate the formation mechanism of group identity among Ultimate Frisbee club members. Specifically, this research aims to achieve three objectives. First, it seeks to identify the key factors involved in the formation of group identity among club members and to explore the internal correlations among these factors. Second, it aims to construct and empirically test a theoretical model detailing the relationship between these factors and group identity. Third, based on the empirical findings, the study provides targeted recommendations for club management practices.

## 2 Theoretical basis and literature review

### 2.1 Interaction ritual chain theory

Proposed by Randall Collins (2004), Interaction Ritual Chain theory provides a microsociological framework for understanding the formation of social solidarity and group identity in face-to-face interactions. Collins, 2004 notes that a successful interaction ritual comprises four core elements: (1) bodily co-presence of two or more individuals in the same physical space; (2) boundaries to outsiders to distinguish members from non-members; (3) a mutual focus of attention, with participants concentrating on a shared activity or object; and (4) a shared emotional mood, where participants experience the same emotions and achieve emotional entrainment. When these conditions are met, the interaction ritual yields a series of collective outcomes: group solidarity, emotional energy, symbols representing the group, and a sense of morality (Collins, 2004). Among these outcomes, emotional energy serves as the central concept in Collins's theoretical framework. Distinct from transient emotional fluctuations, emotional energy is an enduring and stable emotional state manifested as confidence, enthusiasm, and a willingness to take initiative. Collins (2004) posits that individuals are fundamentally motivated to maximize emotional energy, tending to participate in interactions yielding high emotional energy and avoiding those that deplete it. Emotional energy thus operates both as a core product of successful rituals and as a motivating force that encourages individuals to participate in subsequent interactions.

The application of Interaction Ritual Chain theory has expanded into various social contexts, including online communities (Wang and Fu, 2022), sports fan culture (Cottingham, 2012; Fu and Zhang, 2025; Chen and Wang, 2022), and physical activity groups (Shi and Wang, 2023). In sports and leisure contexts, researchers have found that shared emotional experiences generated through collective participation, such as the excitement during intense matches or post-victory celebrations, can generate enduring emotional energy, strengthen the bonds among participants, and foster group identification (Wang and Fu, 2022; Li, 2025). For Ultimate Frisbee clubs, regular ritualized practices such as face-to-face training sessions, competitive matches, and post-game spirit circles provide sufficient conditions for successful interaction rituals. Members repeatedly gather in a shared physical space, maintain clear group boundaries through mechanisms like team uniforms and tryouts, mutually focus on the disc's trajectory and the execution of tactical teamwork, and experience emotional entrainment during critical moments of athletic intensity. These recurring rituals have the potential to generate emotional energy, serving as a mechanism that links collective participation to group identity.

### 2.2 Social exchange theory

Originating from the seminal works of Homans (1958); Thibaut and Kelley (1959), and Blau (1964), Social Exchange Theory provides a complementary theoretical perspective on rational calculation within social relationships. The core premise of this theory posits that individuals participate in social interactions as rational actors seeking to maximize rewards and minimize costs (Cropanzano and Mitchell, 2005). The fundamental distinction between social exchange and economic exchange is that social exchange involves unspecified obligations and relies on trust rather than formal contracts (Blau, 1964).

The norm of reciprocity proposed by Gouldner (1960) serves as the foundational principle of social exchange. This norm requires individuals to reciprocate received benefits, fostering a pattern of mutual obligations that sustains ongoing relationships. When individuals perceive fair reciprocation for their contributions, whether material or non-material, they develop positive attitudes toward their exchange partners and exhibit stronger commitment to the relationship (Cook et al., 2013).

In voluntary organizations like sports clubs, the rewards members derive from participation frequently manifest as relational benefits rather than material compensation. In a service context, Gwinner et al. (1998) identified three types of relational benefits: confidence benefits (feelings of trust and reduced anxiety), Social Benefits (personal recognition and friendship), and special treatment benefits (priority and customized service). Subsequent research has extended these concepts into sports and leisure contexts. Hennig-Thurau et al. (2002) reported that relational benefits were associated with relationship quality, encompassing satisfaction, trust, and commitment. Within sports contexts, Guillet et al. (2002) found that relational benefits perceived by athletes, such as trust from coaches and teammates, can predict their intention to persist in the sport. For Ultimate Frisbee club members, relational benefits may manifest in various forms. Teammates continuously passing the disc to a specific member during crucial points signifies trust in that member's abilities, generating confidence benefits. Social Benefits arise when members feel accepted by the group, establish friendships, and experience a sense of belonging. In clubs without formal incentives, relational benefits may be especially important, and sustained participation may depend substantially on members' perceptions of these social relationships.

### 2.3 Group identity

The concept of group identity is rooted in Social Identity Theory, originally proposed by Tajfel and Turner (1979, 1986). Rooted in Social Identity Theory (Tajfel and Turner, 1979), group identity emphasizes three core components: cognitive categorization, emotional attachment, and evaluative judgment of the group's value.

In organizational and management research, Social Identity Theory has been applied to develop the concept of organizational identification, defined as the perception of oneness with or belongingness to an organization (Ashforth and Mael, 1989). In the field of sports management, researchers have applied these ideas to examine team identification among sports fans (Wann and Branscombe, 1993; Lock and Heere, 2017). Team identification refers to the extent to which fans develop a psychological connection with a sports team, viewing the team's successes and failures as their own.

The measurement of group identity has evolved from a unidimensional to a multidimensional approach. Cameron (2004) proposed a three-factor model comprising cognitive centrality (the prominence of group membership in the self-concept), ingroup affect (positive feelings toward the group), and ingroup ties (perceptions of similarity and connection with other members). Leach et al. (2008) developed a hierarchical model distinguishing between self-definition (similarity and self-stereotyping) and self-investment (solidarity, satisfaction, and centrality). In a sports context, Sutcliffe et al. (2023) validated the Social Identity Questionnaire for Sport-Parents (SIQS-P), encompassing cognitive, affective, and evaluative dimensions. Existing research primarily focuses on fans or consumers of professional sports. Members of lifestyle sports clubs like Ultimate Frisbee play a different role: they are active co-creators of the group experience rather than spectators. Their identification involves emotional attachment and cognitive belonging, while also potentially encompassing active participation and a sense of shared ownership. This characteristic warrants context-specific investigation.

### 2.4 Literature review and research gaps

Existing research on group identity in sports and leisure contexts has made significant progress, yet three prominent research gaps remain.

First, regarding the research context, the majority of studies focus on traditional sports organizations, professional sports teams, or online communities. The context of lifestyle sports clubs, where members are active participants rather than passive consumers, formal authority structures are minimized, and participation is entirely voluntary, remains underexplored. With unique characteristics such as player self-officiating, mixed-gender rosters, and the “Spirit of the Game,” Ultimate Frisbee clubs provide an ideal field setting to examine the formation of group identity in scenarios characterized by high interaction frequency and low institutional control.

Second, concerning theoretical perspectives, previous studies have often adopted an emotional perspective (based on Interaction Ritual Chain Theory) or a rational perspective (based on Social Exchange Theory) in isolation. Few studies have integrated both perspectives into a single framework to examine the joint operation of emotional and rational mechanisms in the formation of group identity. Given that individual participation in a group involves both emotional experiences and cognitive calculations, a dual-pathway model may offer a more comprehensive explanation.

Third, in terms of research methodology, most studies rely on either solely qualitative or solely quantitative methods. While qualitative research can provide rich contextual understanding, it may lack generalizability; conversely, quantitative research can test predetermined constructs but might overlook context-specific dimensions. A mixed-methods approach combining inductive exploration with deductive verification is well suited to investigating emerging phenomena. Such designs remain rare in this field.

This study adopts a two-stage mixed-methods approach. The first stage utilizes a Grounded Theory approach to inductively explore the formation mechanisms of group identity among Ultimate Frisbee club members, identifying key factors and their internal correlations. The second stage develops a contextualized measurement tool and employs structural equation modeling to quantitatively test the theoretical model, focusing on the mediating roles of emotional energy and relational benefits.

## 3 Study 1: a grounded theory model of group identity formation

Study 1 involves an inductive exploration of the mechanisms underlying group identity formation within Ultimate Frisbee clubs. The grounded theory approach (Corbin and Strauss, 2015) is well suited to the novelty of this phenomenon and the current lack of specialized theoretical frameworks for this context. This approach integrates the unique characteristics of Ultimate Frisbee clubs into the analytical framework. Study 1 addresses the following questions: what are the key factors in the formation of group identity among Ultimate Frisbee club members, and what are the interconnections among these factors?

### 3.1 Research methodology

#### 3.1.1 Grounded theory approach and methodological justification

Grounded theory was selected because the study aimed to develop a context-specific theoretical account of group identity formation in a lifestyle sport setting for which no established explanatory framework existed, rather than to test a pre-existing model (Glaser and Strauss, 1967; Corbin and Strauss, 2015). We adopted the systematic (Straussian) variant of grounded theory because it provides explicit coding procedures and permits prior theory to function as sensitizing concepts without determining the emerging categories (Corbin and Strauss, 2015).

Theoretical saturation was assessed with the five reserved transcripts: no new concepts or relationships emerged, indicating that the main categories and their properties were saturated (Corbin and Strauss, 2015). We acknowledge that saturation is not absolute and that additional interviews could reveal further nuance.

#### 3.1.2 Sampling and participant selection

Theoretical sampling was operationalized through purposive selection of participants from clubs in Shanghai, Hangzhou, and Shenzhen, with deliberate diversity in playing experience (from less than 1 year to 14 years) and organizational role (general members, captains, and club founders). This design allowed the sample to cover a broad range of perspectives relevant to the formation of group identity.

#### 3.1.3 Theoretical sensitization

Interaction Ritual Chain Theory and Social Exchange Theory informed the study as sensitizing frameworks from the outset. Collins's (2004) ritual ingredients and the relational-benefits typology guided initial questions about shared practices, emotional experiences, trust, and social acceptance. During open coding, concepts were labeled in participants' own words, and the coding scheme remained open to unanticipated categories; prior theory was treated as an analytic resource rather than a template. Integration of the emergent categories with the two theories occurred mainly during selective coding and interpretation. The qualitative phase was therefore partially theory-informed, and we report this explicitly to maintain methodological transparency.

#### 3.1.4 Data collection

Data collection occurred through semi-structured interviews conducted between April and May 2025. These sessions utilized both online and offline formats, with each interview lasting between 45 and 60 m. All interviews were recorded following the attainment of informed consent from participants and subsequently transcribed verbatim. The interviews produced approximately 88,000 words of textual data. The interview guide focused on experiences related to joining, participating in, and identifying with the club. Discussions explored initial entry experiences, significant interaction moments, senses of belonging, relationships with teammates, and motivations for continued participation or withdrawal.

To supplement the interview data, the first author conducted participant observations during club training and competitions, recording field notes on interaction patterns, ritualized behaviors, and group dynamics. These observations added contextual depth and supported triangulation of the interview findings. Out of 22 interview transcripts, 17 were utilized for initial coding and model construction, while the remaining five were reserved for testing theoretical saturation (see Appendix A for participant demographics).

#### 3.1.5 Data analysis

Data analysis followed the three-step coding procedure of systematic grounded theory: open coding, axial coding, and selective coding (Corbin and Strauss, 2015). The MAXQDA 2020 software facilitated systematic coding and the recording of analytical memos. The primary author performed the initial coding and engaged in regular discussions with collaborators regarding emerging categories to enhance analytical rigor. Open coding involved a line-by-line analysis of the interview transcripts. Concepts were labeled using the participants' own words to maintain proximity to the raw data. Through constant comparison, similar concepts were grouped into initial categories. This process yielded 90 initial concepts. Axial coding focused on identifying relationships between the categories generated during open coding. The paradigm model—comprising conditions, phenomena, context, intervening factors, action/interaction, and consequences—organized the categories into higher-order themes and sub-categories. This stage resulted in 11 sub-categories and 4 main categories. Selective coding integrated and refined the core categories into a coherent theoretical framework. The core category, defined as the formation mechanism of group identity among Ultimate Frisbee club members, emerged through the development of a storyline describing the central process between main categories. Theoretical saturation was tested using the five reserved interview transcripts. No new concepts or relationships emerged during the coding of these additional interviews, indicating that the categories reached saturation. Throughout analysis, MAXQDA 2020 was used to manage coding, memos, and category relationships. Coding decisions and evolving interpretations were documented in analytical memos, and the researchers revisited provisional category labels through constant comparison. The credibility of the analysis was supported by triangulation between interview data and participant observation, systematic memo writing, and the theoretical saturation test using the five reserved transcripts.

### 3.2 Research findings

The grounded theory analysis indicated that the formation of group identity among Ultimate Frisbee club members involves three core factors: interaction rituals, emotional energy, and relational benefits. These factors jointly contributed to group identity formation. These elements are organized into a dual-pathway model consisting of an affective pathway (interaction rituals → emotional energy → group identity) and a rational pathway (interaction rituals → relational benefits → group identity). The following sections detail each category and its interconnections.

#### 3.2.1 Open coding

Open coding produced 90 initial concepts from the raw data. Appendix B provides an example of the coding process, illustrating how raw statements were labeled and conceptualized. Concepts such as wearing team uniforms, shouting slogans, sideline support, trust, and teammates being like family reflected participants' experiences.

#### 3.2.2 Axial coding

Through axial coding, the 90 initial concepts were categorized into 11 sub-categories, which were further abstracted into 4 main categories: interaction rituals, emotional energy, relational benefits, and group identity. Table 1 presents the category structure and definitions.

**Table 1:** Axial coding category extraction.

Main category	Subcategory	Category connotation
**Interaction rituals**	Co-presence	A micro-situation in which members are physically co-present and interact in real-time during club-related activities.
	Group boundaries	Symbolic markers used by members to distinguish between in-group and out-group.
	Mutual focus	Shared events or moments that highly concentrate members' attention during the ritual process.
	Emotional entrainment	Emotional states that members collectively experience and mutually reinforce in interaction rituals.
**Emotional energy**	Vigor energy	A positive state of feeling energetic and more confident that members experience after participating in club activities.
	Enthusiasm energy	A sense of anticipation and engagement that members develop toward club activities.
**Relational benefits**	Trust benefits	The psychological benefit of being trusted by teammates.
	Social benefits	The psychological benefit of being accepted by the group, along with the interpersonal relationships thus established.
**Group identity**	Cognitive identity	The cognitive process through which individuals categorize themselves as members of the club.
	Affective identity	The emotional attachment individuals develop toward the club.
	Evaluative identity	The positive value judgment individuals assign to their club membership and the group characteristics it represents.

#### 3.2.3 Selective coding and the dual-pathway model

Selective coding integrated the main categories into a coherent storyline. The core phenomenon is the formation mechanism of group identity among Ultimate Frisbee club members. The storyline is summarized as follows.

Upon joining a club, members participate in recurring interaction rituals characterized by physical co-presence, Group Boundaries, mutual focus, and emotional entrainment. These rituals are associated with two types of outcomes, which serve as parallel pathways toward group identity.

The affective pathway involves interaction rituals linked with the generation of emotional energy. This energy manifests as vitality (feelings of vigor and confidence) and enthusiasm (anticipatory excitement). This enduring positive affect may bind members' subjective experiences to the club and promote emotional attachment, thereby contributing to group identity formation.

Simultaneously, the rational pathway involves interaction rituals associated with relational benefits, specifically Trust Benefits (being trusted by teammates) and Social Benefits (being accepted and establishing friendships). Members perform cognitive evaluations of these benefits, viewing them as rewards for continued participation. This rational calculation may facilitate the internalization of club membership into the self-concept and thereby contribute to group identity through cognitive and evaluative dimensions. These two pathways may operate in parallel and reinforce one another. Figure 1 illustrates the theoretical model derived from the grounded theory analysis.

**Figure 1:**
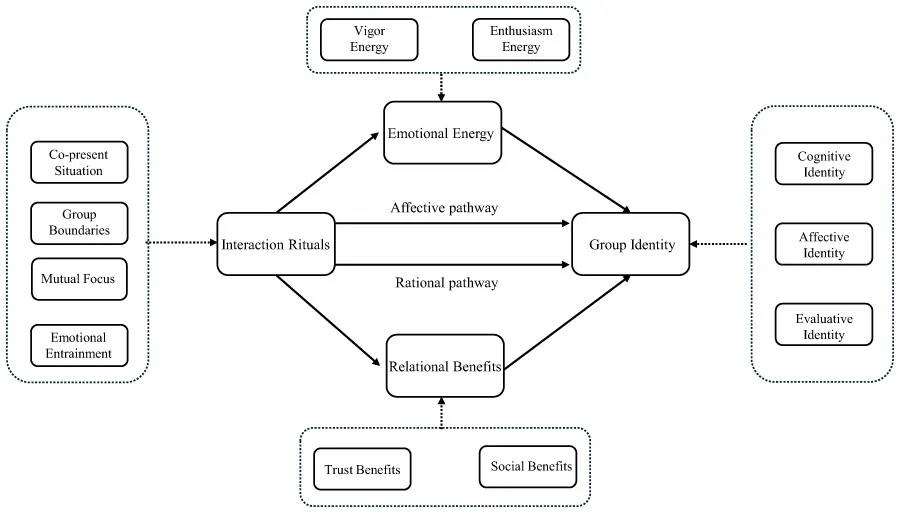
Model of group identity formation based on grounded theory.

#### 3.2.4 Theoretical saturation test

Coding the five reserved interview transcripts served to verify theoretical saturation. This analysis revealed no new concepts or relationships beyond the initial coding categories. The existing framework encompasses the experiences detailed in the saturation test interviews, suggesting that the model reached theoretical saturation.

### 3.3 Theoretical model

#### 3.3.1 Interaction rituals: the starting point of group identity formation

Through a procedural grounded theory analysis, this study indicated that, in Ultimate Frisbee clubs, interaction rituals manifest as embodied practices enacted by members during regular training sessions and competitions across four dimensions: co-presence, group boundaries, mutual focus, and emotional entrainment.

##### 3.3.1.1 Co-presence

In Ultimate Frisbee clubs, this form of physical assembly is characterized by high frequency, extended duration, and cross-contextual presence. Members showed high institutional discipline by dedicating full days to training and closely following attendance rules, which transformed daily participation into a stable collective practice. Co-presence was also evident in the collective overcoming of physical environmental challenges. As one participant noted: “During the typhoon, so many people were still willing to come train together, and no one said they wanted to leave early” (Interview 9–6). The act of gathering despite adverse weather and physical barriers itself sent a strong ritual signal, transforming physical assembly into a shared behavioral framework.

##### 3.3.1.2 Group Boundaries

Group boundaries did not emerge from physical isolation but were constructed through exclusive visual symbols, institutional selection, and shared cognitive practices. Clubs establish spatial distinction through customized team uniforms, which serve as exclusionary markers of identity rather than mere athletic apparel. This visual boundary was reinforced by institutionalized selection mechanisms, such as tryouts and trial periods, which ensured that participants aligned with the group's athletic and normative expectations. Highlighting this intersection of visual and institutional boundaries, one member stated: “Our uniforms are not sold to outsiders. Only after you join this club and pass the assessment can you buy this uniform” (Interview 8–10). Furthermore, clubs reinforced internal cognitive boundaries through exclusive ritualized behaviors, such as pre-game cheers and proprietary tactical terminology, creating a closed communication system that distinguished “insiders” from external bystanders.

##### 3.3.1.3 Mutual focus

Mutual focus translated physical co-presence into psychological resonance through intensive concentration on shared competitive goals, tactical execution, and reciprocal attention. During high-speed transitions, members achieved a high degree of cognitive synchronization by focusing simultaneously on the trajectory of the disc and their teammates' action signals. This shared orientation reduced individual distractions and fostered a task-focused environment. As one participant captured this collective state: “We never give up until the frisbee hits the ground at the last second. At that moment, everyone's eyes are fixed on that one disc” (Interview 9–3). Through this reciprocal cycle of attention, each individual becomes an active node within the collective interaction network, reducing the need for verbal communication.

##### 3.3.1.4 Emotional entrainment

Emotional entrainment emerged through emotional contagion, affective resonance, and collective effervescence. Rather than relying on an abstract psychological contract, this shared emotional state was forged through micro-level interactions under competitive pressure. During intense sessions, individual dedication rapidly spread, synchronizing the team's affective state without the need for verbal encouragement. This emotional synchronization often peaks during critical match moments, propelling the group toward collective effervescence, whether celebrating a victory or sharing the burden of defeat. As one participant vividly recalled during a high-pressure defensive stand: “I felt like the heartbeat of everyone on the field was synchronized. I'm not exaggerating; I could genuinely feel that everyone around me was completely united in effort” (Interview 21–6). Through these recurring moments of affective resonance, members reinforced their emotional connection to the group.

#### 3.3.2 Emotional energy: the affective driver of group identity formation

In Ultimate Frisbee clubs, emotional energy manifests in two primary dimensions: vigor energy and enthusiasm energy. Vigor energy refers to the cognitive state of alertness and mental agility gained through sustained ritual participation, which often counters the physical fatigue of the sport. Rather than depleting their energy, members experience a positive spillover effect into their daily lives. As one participant noted: “Before, I would just scroll on my phone... now, training two or three times a week, I actually feel like I've been ‘activated,' and I can concentrate better at work” (Interview 6–13). Concurrently, enthusiasm energy captures an individual's deep anticipation and proactive engagement with club activities. This emotional investment transforms club participation from an external schedule into an internalized drive, where members willingly sacrifice leisure time, such as sleeping in, for the sport (Interview 11–17). Through sustained vigor and enthusiasm, emotional energy became a strong affective driver encouraging members to return to the ritual. Members no longer required external encouragement but are drawn to the training field, to teammates, and to the next ritual by their intrinsic enthusiasm. This transformation illustrates the role of emotional energy as a sustained motivating force.

#### 3.3.3 Relational benefits: the rational calculus of group identity formation

Beyond core athletic activities, members accumulated substantial relational benefits through sustained interaction, centered primarily on trust and social acceptance. In the context of Ultimate Frisbee, disc possession functions as a symbolic expression of trust. Members rationally evaluate these Trust Benefits based on real-time game dynamics rather than verbal promises. Highlighting this conditional trust, one participant explained: “Trust is built like this: if I pass to you 10 times in a game and you rarely make mistakes, then trust emerges quickly. If you drop it two or three times, then that trust disappears, and it is very difficult to regain” (Interview 9–17). Parallel to trust, members accumulate Social Benefits through an inclusive, non-judgmental atmosphere that fosters psychological safety. The group's undifferentiated treatment of newcomers and focus on constructive problem-solving rather than blaming (Interview 13–14) provided members with a sense of social security, serving as a rational incentive for continued participation.

#### 3.3.4 Group identity: the identity transformation from “I” to “We”

Group identity within Ultimate Frisbee clubs is a multidimensional psychological construction, manifesting as deep internalization across cognitive, affective, and evaluative dimensions. Cognitively, members undergo a shift in perspective, moving from an external view of “this team” to an internalized “our team,” marking the crossing of symbolic psychological boundaries. As one member expressed: “I feel that I belong to this team. No matter what others say, this is where I identify myself” (Interview 7–15). Affectively, this cognitive categorization evolves into deep emotional attachment, where the club transforms into an emotional community and teammates are viewed as “family” (Interview 18–14). Finally, this identity is externalized through evaluative judgments, where members set ambitious collective goals and actively uphold the club's superiority through horizontal comparisons with rival teams. One participant demonstrated this ingroup bias by contrasting their team's receptive culture with the arrogance of a competitor (Interview 1–12), reflecting the internalization of group pride.

#### 3.3.5 Pathways to group identity formation: the dual drivers of affect and rationality

Through the constant comparative analysis of the core categories, this study identifies that group identity formation among Ultimate Frisbee club members operates through a dual-pathway mechanism, rather than a single linear trajectory.

The first is an affective pathway driven by the accumulation of emotional energy. Consistent with Collins's theoretical logic, high-frequency interaction rituals appeared to generate collective effervescence, which sustained members' emotional energy (vigor and enthusiasm). This sustained emotional vitality may reduce individual psychological boundaries and facilitate affective attachment and identity internalization. As one participant recalled: “There are those thrilling moments when we run together on the field, sharing hardships and joys for one disc. In those moments, our hearts are closest” (Interview 8–18).

The second is a rational pathway driven by the calculation of relational benefits. Aligning with social exchange theory, members concurrently evaluate the intangible social returns (trust, acceptance, and security) against their investments of time and effort. When members perceived this social exchange as equitable, rational belonging could emerge. A participant's self-analysis illustrates this instrumental logic: “If I train very diligently and perform well, but my input is not proportionate to my returns, then I would question what the point is of staying in this team” (Interview 7–6).

Ultimately, these two pathways operate in parallel and reinforce one another. As illustrated in Figure 1, interaction rituals serve as the starting point, facilitating the parallel accumulation of emotional energy and relational benefits, both of which may jointly contribute to group identity formation in a voluntary sports organization.

## 4 Study 2: scale development and model testing using SEM

Building upon the qualitative findings from Study 1, Study 2 aims to provide a quantitative validation of the dual-pathway model regarding group identity formation. Specifically, this phase involves developing measurement scales for the core constructs and employing structural equation modeling (SEM) to examine the hypothesized relationships. This study also investigates the mediating roles of emotional energy and relational benefits.

### 4.1 Research hypotheses

#### 4.1.1 Interaction rituals and group identity

Interaction ritual chain theory posits that successful interaction rituals, which are characterized by physical co-presence, group boundaries, a mutual focus of attention, and a shared emotional mood, generate group solidarity and collective identity (Collins, 2004). Within Ultimate Frisbee clubs, members regularly participate in ritualized activities such as training sessions, matches, and post-game spirit circles. These rituals provide opportunities for members to develop a sense of belonging and to internalize the club into their self-concept. Accordingly, this study proposes the following hypothesis:

- H1: Interaction rituals relate positively to the group identity of members.

#### 4.1.2 The mediating role of emotional energy

According to Collins (2004), emotional energy represents the primary outcome of successful interaction rituals. It manifests as an enduring emotional state characterized by confidence, enthusiasm, and initiative. As members experience recurring rituals, they accumulate emotional energy, which subsequently facilitates group solidarity and identity. Empirical studies have provided evidence that emotional energy may function as a mediator between interaction rituals and identity-related outcomes (Xue, 2021; Wang and Fu, 2022). Consequently, the following hypotheses are proposed:

- H2: Interaction rituals are positively associated with emotional energy.
- H3: Emotional energy relates positively to group identity.
- H4: Emotional energy serves as a mediator between interaction rituals and group identity.

#### 4.1.3 The mediating role of relational benefits

Social exchange theory suggests that individuals participate in social interactions with the expectation of receiving rewards that exceed the associated costs (Blau, 1964; Cropanzano and Mitchell, 2005). In the context of sports clubs, relational benefits, such as trust from teammates and social acceptance, may function as rewards that strengthen members' attachment to the group (Guillet et al., 2002). When members perceive that their participation yields trust and Social Benefits, they are more likely to incorporate club membership into their self-concept. Therefore, the following hypotheses are proposed:

- H5: Interaction rituals are positively related to relational benefits.
- H6: Relational benefits relate positively to group identity.
- H7: Relational benefits function as a mediator between interaction rituals and group identity.

Figure 2 presents the theoretical model including all hypothesized paths.

**Figure 2:**
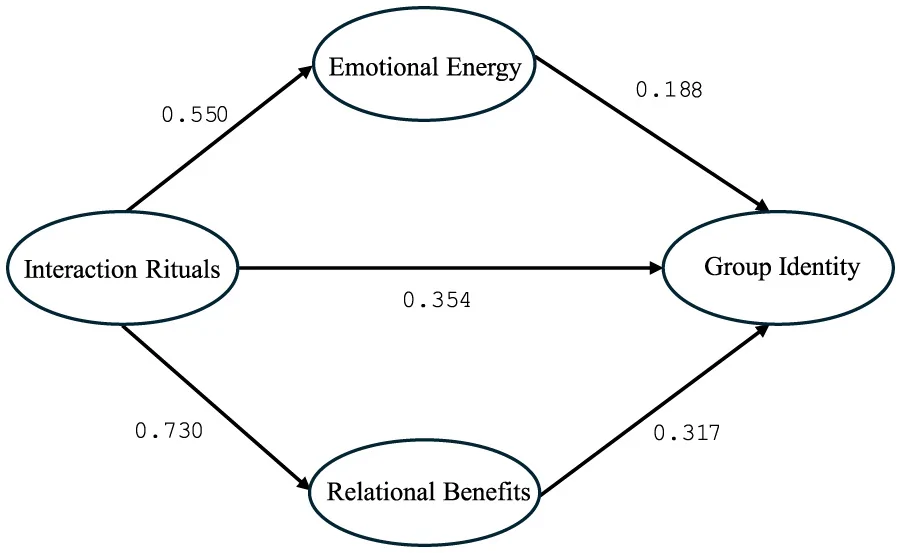
Theoretical model of group identity formation among ultimate frisbee club members.

### 4.2 Research methodology

#### 4.2.1 Scale development

Content validity was established through three procedures. First, items were generated from the categories and participants' own expressions identified in Study 1, ensuring contextual relevance. Second, items were adapted from established measures (Cameron, 2004; Leach et al., 2008; Hennig-Thurau et al., 2002; Guillet et al., 2002; Sutcliffe et al., 2023) and reworded for the Ultimate Frisbee context. Third, a focus group of six Ultimate Frisbee club members (three men and three women) reviewed the initial item pool for clarity, relevance, comprehensiveness, and redundancy; ambiguous or overlapping items were rephrased or merged.

The development of the measurement scales followed established procedures for construct development within management research (Lewis et al., 2005). This process consisted of four distinct stages: (1) generating initial items from qualitative data and existing literature; (2) refining these items through focus group interviews; (3) conducting a small sample pilot test to evaluate reliability and validity; and (4) implementing the formal survey.

Items for interaction rituals originated from the four dimensions identified in Study 1, which included physical co-presence, Group Boundaries, mutual focus, and emotional entrainment. These dimensions produced 17 initial items. Items for emotional energy were adapted from existing scales (Xue, 2021) and integrated with qualitative findings to form 4 items. Relational benefit items were adapted from previous research regarding relational benefits in service contexts (Hennig-Thurau et al., 2002; Guillet et al., 2002) and modified for the Ultimate Frisbee context, yielding 7 initial items. Items for group identity were adapted from established scales (Cameron, 2004; Leach et al., 2008; Sutcliffe et al., 2023) and refined based on qualitative findings, resulting in 10 initial items.

A focus group consisting of six Ultimate Frisbee club members (three men and three women) reviewed the initial items. The participants evaluated the clarity of each item, its relevance to the club context, and its overall comprehensiveness. Based on the feedback provided, the researchers merged semantically similar items, rephrased ambiguous wording, and adjusted the dimensional structure to enhance logical consistency. This process produced a refined set of items including 15 items for interaction rituals across four dimensions, 4 items for emotional energy in a single dimension, 6 items for relational benefits across two dimensions, and 9 items for group identity across three dimensions (Appendix C). All items used a 5-point Likert scale ranging from 1 (strongly disagree) to 5 (strongly agree).

#### 4.2.2 Pilot test

A pilot test was conducted with a sample of 110 Ultimate Frisbee club members in Shanghai to evaluate the reliability and validity of the measurement scales. Participants completed a questionnaire containing 34 items, and the data underwent analysis using SPSS 27.0.

In the pilot test (*N* = 110), items were retained when they met all of the following criteria: corrected item-total correlation above 0.50, primary factor loading above 0.50, no substantial cross-loading (loadings on other factors below 0.40), and a meaningful contribution to the theoretical structure. Principal component analysis with rotation was used, and factors were retained when their eigenvalues exceeded 1.0 and the solution was interpretable. After deleting interaction-ritual items I7, I8, and I12, the four-factor solution explained 86.952% of the total variance, with KMO = 0.783.

The researchers calculated the Cronbach's alpha coefficients for each construct. The results indicated good reliability for interaction rituals (alpha = 0.904), emotional energy (alpha = 0.944), relational benefits (alpha = 0.877), and group identity (alpha = 0.883). All corrected item-total correlation (CITC) values exceeded 0.5, which suggests adequate internal consistency.

Exploratory factor analysis (EFA) was performed to examine the factor structure. For interaction rituals, the analysis indicated a four-factor structure corresponding to physical co-presence, Group Boundaries, mutual focus, and emotional entrainment. This structure accounted for 85.73% of the total variance. Following the removal of items with cross-loadings or low factor loadings, 12 items remained. Relational benefits showed a two-factor structure comprising Trust Benefits and Social Benefits, which explained 82.46% of the total variance. Group identity showed a three-factor structure consisting of cognitive identity, affective identity, and evaluative identity, which explained 85.53% of the total variance. Emotional energy showed a single factor structure with factor loadings exceeding 0.90. Table 2 presents the final measurement scale.

**Table 2:** Final measurement scale.

Construct	Dimension	Item
Interaction ritual	Co-presence	During competitions, I travel to the venue with my teammates.
		I regularly participate in club training.
		I persist in training even in bad weather.
	Group boundaries	I understand the club's tactical terminology.
		I participate in the club's tryouts.
		The club training I attend is not open to the public.
	Mutual focus	I respond when a teammate makes a good play.
		I listen attentively when the coach explains tactics.
		When making a cut, I can sense my teammates' gaze.
	Emotional entrainment	I resonate with my teammates during the post-game circle.
		My teammates' training status affects me.
		During games, I stay in sync with the team's rhythm.
Emotional energy		The post-game circle energizes me.
		Participating in club activities boosts my self-confidence.
		Participating in club activities gives me vitality.
		I look forward to the club's next training session.
Relational benefits	Trust benefits	Teammates trust me enough to pass me the disc during crucial points.
		I can feel my teammates' trust in me.
		I have good chemistry with my teammates.
	Social benefits	I have made friends within the club.
		Teammates actively communicate with me.
		Teammates care about my well-being.
Group identity	Cognitive identity	Whether winning or losing, I am a member of the club.
		When introducing myself, I say “our team” rather than “their team.”
		The club is a part of my life.
	Affective identity	The club gives me a sense of belonging, like “home.”
		I am proud to be a member of this club.
		Even when not training, I still think about this club.
	Evaluative identity	I believe my club is better than other clubs.
		My view of the club is positive.
		I feel offended when outsiders criticize the club.

#### 4.2.3 Formal survey

The formal survey took place from October 15 to November 14, 2025. Data collection involved both online and offline channels. The online portion utilized social media groups within Ultimate Frisbee clubs and applied snowball sampling techniques. The offline portion consisted of paper questionnaires distributed during field visits to training locations in Shanghai. The study distributed 421 questionnaires and recovered 376. Following the exclusion of invalid responses, 332 valid questionnaires remained. This corresponds to an effective response rate of 78.9%. The final sample included 228 responses from online channels and 104 from offline channels.

#### 4.2.4 Statistical power and common method bias

A *post hoc* Monte Carlo power analysis (Muthén and Muthén, 2002) was conducted using the standardized estimates from the final model, the observed correlations among the latent constructs, and the final sample size (*N* = 332). Across 5,000 simulated replications, power to detect the indirect effect through emotional energy was 0.98 and through relational benefits was 0.99 at alpha = 0.05.

Because all quantitative constructs were measured with self-report items at a single time point, common method bias was assessed with Harman's single-factor test (Podsakoff et al., 2003). All items were entered into an unrotated exploratory factor analysis; the first factor accounted for 32.46% of the total variance, below the 50% threshold. Procedural remedies included anonymous questionnaires, confidentiality assurances, and instructions emphasizing that there were no right or wrong answers.

### 4.3 Research results

#### 4.3.1 Measurement model evaluation

Confirmatory factor analysis (CFA) using AMOS 24.0 served to evaluate the measurement properties of the scales. As shown in Table 3, all standardized factor loadings exceeded the 0.60 threshold (*p* < 0.05). The composite reliability (CR) values for all constructs ranged from 0.870 to 0.922, well above the recommended 0.70 benchmark. Additionally, the average variance extracted (AVE) values ranged from 0.675 to 0.796, satisfying the minimum requirement of 0.50 (Fornell and Larcker, 1981). These results provide support for convergent validity across the measurement model.

**Table 3:** Measurement model reliability and validity.

Construct	Dimension	Items	Factor loadings	CR	AVE
Interaction ritual	Co-presence	I1	0.887	0.899	0.747
		I2	0.833		
		I3	0.872		
	Group boundaries	I4	0.896	0.922	0.796
		I5	0.901		
		I6	0.88		
	Mutual focus	I7	0.876	0.898	0.747
		I8	0.843		
		I9	0.873		
	Emotional entrainment	I10	0.855	0.875	0.7
		I11	0.862		
		I12	0.792		
Emotional energy	-	EE1	0.81	0.893	0.675
		EE2	0.802		
		EE3	0.86		
		EE4	0.814		
Relational benefits	Trust benefits	R1	0.868	0.89	0.73
		R2	0.830		
		R3	0.865		
	Social benefits	R4	0.783	0.87	0.691
		R5	0.868		
		R6	0.840		
Group identity	Cognitive identity	G1	0.850	0.902	0.754
		G2	0.893		
		G3	0.861		
	affective identity	G4	0.907	0.885	0.720
		G5	0.787		
		G6	0.848		
	Evaluative identity	G7	0.847	0.894	0.737
		G8	0.838		
		G9	0.889		

Discriminant validity was examined using the Fornell-Larcker criterion (Fornell and Larcker, 1981). Table 4 reports the square root of the second-order construct-level AVE on the diagonal and the manifest inter-construct correlations off the diagonal; all diagonal values exceeded the corresponding correlations. Dimension-level AVE and CR values are reported in Table 3.

**Table 4:** Discriminant validity test.

Variable	Square root of AVE	1	2	3	4
Interaction ritual	0.680	0.680			
Emotional energy	0.822	0.415	0.822		
Relational benefits	0.728	0.472	0.403	0.728	
Group identity	0.700	0.479	0.413	0.449	0.700

#### 4.3.2 Structural model and hypothesis testing

Table 5 reports the correlations among the study variables. Interaction rituals were positively correlated with emotional energy, relational benefits, and group identity, providing preliminary support for the hypothesized relationships. The structural model exhibited acceptable fit: chi-square/df = 1.280, RMSEA = 0.029, NFI = 0.927, RFI = 0.920, IFI = 0.983, TLI = 0.981, and CFI = 0.983 (Hu and Bentler, 1999). As shown in Table 6, all direct paths were statistically significant, supporting H1, H2, H3, H5, and H6. The model explained 30.2% of the variance in emotional energy, 53.2% of the variance in relational benefits, and 54.5% of the variance in group identity. Table 6 reports AMOS bias-corrected 95% bootstrap confidence intervals for all structural paths.

**Table 5:** Correlation analysis of research variables.

Construct	1	2	3	4	5	6	7	8	9	10	11	12
Interaction ritual	1											
Co-presence	0.749	1										
Group boundaries	0.749	0.341	1									
Mutual focus	0.733	0.421	0.409	1								
Emotional entrainment	0.767	0.492	0.466	0.365	1							
Emotional energy	0.415	0.331	0.254	0.350	0.311	1						
Relational benefits	0.472	0.322	0.365	0.354	0.375	0.403	1					
Trust benefits	0.424	0.329	0.292	0.294	0.359	0.343	0.860	1				
Social benefits	0.387	0.224	0.334	0.314	0.285	0.349	0.856	0.473	1			
Group identity	0.479	0.355	0.350	0.377	0.354	0.413	0.449	0.390	0.382	1		
Cognitive identity	0.307	0.235	0.234	0.220	0.232	0.272	0.305	0.272	0.252	0.772	1	
Affective identity	0.402	0.300	0.266	0.360	0.280	0.329	0.392	0.316	0.357	0.795	0.403	1
Evaluative identity	0.429	0.310	0.332	0.314	0.329	0.380	0.371	0.339	0.298	0.815	0.457	0.477

*N* = 332; ^**^p < 0.01.

**Table 6:** Path coefficients and significance levels.

Path	Unstandardized coefficient	Standardized coefficient	S.E.	C.R.	*P*	95% BC CI	*R* ^2^
Interaction ritual → Group identity	0.283	0.354	0.116	2.427	0.015	[0.013, 0.596]	0.545
Interaction ritual → Emotional energy	0.755	0.55	0.105	7.198	^***^	[0.552, 0.997]	0.302
Interaction ritual → Relational benefits	0.758	0.73	0.104	7.315	^***^	[0.552, 0.980]	0.532
Emotional energy → Group identity	0.109	0.188	0.045	2.441	0.015	[0.000, 0.205]	
Relational benefits → Group identity	0.244	0.317	0.106	2.305	0.021	[0.005, 0.599]	

^***^indicates\(*p* < 0.001\). *R*^2^ values are squared multiple correlations of the endogenous latent variables: Emotional Energy = 0.302, Relational benefits = 0.532, and Group identity = 0.545.

#### 4.3.3 Mediation effect test

To evaluate the mediation effects related to H4 and H7, the study used the bootstrap method with 2,000 resamples and bias-corrected confidence intervals (Preacher and Hayes, 2008). Table 7 presents the indirect effects alongside the 95% confidence intervals.

**Table 7:** Mediation effect analysis of emotional energy and relational benefits.

Path	Standardized effect	Proportion of effect	SE	Lower	Upper	*P*
indA1	0.103	14.97%	0.050	0.005	0.198	0.043
indA2	0.231	33.58%	0.136	0.001	0.552	0.048
Direct effect	0.354	51.45%	0.170	0.010	0.687	0.046
Total effect	0.688	100%	0.069	0.546	0.816	0.001

indEE: interaction rituals → emotional energy → group identity; indRB: indirect effect of interaction rituals → relational benefits → group identity.

Table 7 reports standardized indirect effects with 95% bias-corrected bootstrap confidence intervals based on 2,000 resamples. Emotional energy mediated the relationship between interaction rituals and group identity [standardized indirect effect = 0.103, 95% CI (0.005, 0.198)], and relational benefits also mediated this relationship [standardized indirect effect = 0.231, 95% CI (0.001, 0.552)]. The direct effect remained statistically significant [standardized = 0.354, 95% CI (0.010, 0.687)], indicating partial mediation. The standardized total effect was 0.688, decomposed as 14.97% through emotional energy, 33.58% through relational benefits, and 51.45% direct.

## 5 Discussion

This study extends the understanding of group identity formation within voluntary sports organizations by empirically testing a dual-pathway model. The following sections contextualize the concurrent operation of the affective pathway (emotional energy) and the relatively stronger rational pathway (relational benefits) within existing theoretical frameworks.

### 5.1 The role of interaction rituals in group identity formation

The finding that interaction rituals were positively associated with group identity (H1) is consistent with interaction ritual chain theory (Collins, 2004). This theory posits that successful interaction rituals generate group solidarity and collective identity. Within Ultimate Frisbee clubs, several factors create conditions conducive to identity formation. These factors include physical co-presence during training and matches, clear boundaries maintained through team uniforms and selection mechanisms, a mutual focus on the trajectory of the disc and tactical coordination, and emotional entrainment during critical moments of athletic intensity.

These findings extend the scope of interaction ritual research within sports contexts. Previous studies on sports fans (Cottingham, 2012; Fu and Zhang, 2025; Chen and Wang, 2022) suggest that rituals such as chanting and collective celebrations facilitate identity formation among spectators. However, this study suggests that such effects may be more pronounced among participants. Unlike fans observing from the sidelines, club members immerse themselves deeply in the ritual and experience the process from within. This sense of immersion likely strengthens the influence of rituals on identity formation. This finding suggests that active participation may amplify the ritual mechanisms discussed by Collins (2004).

### 5.2 The mediating role of emotional energy

The mediating role of emotional energy between interaction rituals and group identity (H2, H3, H4) is consistent with the central assertion of Collins (2004) that emotional energy represents the critical mechanism linking rituals to group solidarity. Members of Ultimate Frisbee clubs accumulate emotional energy through recurring participation in rituals. This energy manifests as vitality, such as feelings of vigor and confidence, and enthusiasm, such as anticipatory excitement. These states may subsequently foster identification with the club.

This finding is consistent with existing research in sports and leisure contexts. Xue (2021) observed that emotional energy mediates the relationship between community interaction rituals and fan loyalty. Wang and Fu (2022) reported that emotional energy accumulated in online fitness communities was associated with a stronger sense of belonging among members. The current study extends these findings to offline settings involving active athletic participation. The results suggest that emotional energy functions as a mediator within face-to-face sports clubs.

The indirect effect of emotional energy accounts for 14.97% of the total effect. Although statistically significant, this contribution was smaller than that of relational benefits. This suggests that emotional experiences contribute to identity formation but do not represent the primary mechanism in this specific context. One possible explanation involves the nature of Ultimate Frisbee clubs as voluntary organizations that attract individuals seeking meaningful social connections. For these individuals, the rational evaluation of Relational Benefits may take precedence over emotional experiences.

### 5.3 The mediating role of relational benefits

The mediating role of relational benefits between interaction rituals and group identity (H5, H6, H7) aligns with social exchange theory (Blau, 1964; Cropanzano and Mitchell, 2005). This theory emphasizes rational calculation and reciprocal obligations within social relationships. Club members appeared to view Trust Benefits, such as being trusted by teammates, and Social Benefits, such as being accepted and establishing friendships, as valuable returns for their investment of time and effort. When members perceive these rewards as equitable, they develop positive attitudes toward the club and internalize their membership into their self-concept.

This finding corresponds with research on relational benefits in sports. Guillet et al. (2002) found that the trust and recognition perceived by handball players from coaches and teammates predict their intention to persist in the sport. Hennig-Thurau et al. (2002) reported that relational benefits were associated with relationship quality in service contexts. The present study extends these findings by examining relational benefits as a mediator of identity formation.

At the descriptive level, the standardized indirect effect through relational benefits (0.231) was larger than that through emotional energy (0.103), accounting for 33.58 and 14.97% of the total effect, respectively. Because the data are cross-sectional, we cannot conclude that relational benefits are causally more important than emotional energy. The two mechanisms may operate reciprocally over time: emotional energy generated through rituals may deepen trust and acceptance, and accumulated relational benefits may in turn intensify emotional attachment. Alternative explanations include selection effects, reciprocal causation between identity and perceived benefits, and unmeasured variables such as club tenure, participation frequency, and individual differences. We therefore interpret the observed difference as evidence that relational benefits represent a substantial and statistically significant pathway, and we recommend longitudinal designs to examine temporal precedence and reciprocal dynamics.

These findings connect with the broader social identity approach in sport psychology, which holds that the internalization of group membership underpins motivation, wellbeing, and sustained participation (Rees et al., 2015; Bruner et al., 2014; Benson and Bruner, 2018; Worley and Smith, 2026). Our results complement work on team identity and cohesion in organized sport (Eys et al., 2009; Lock and Heere, 2017; Slater et al., 2013; Bruner et al., 2022) and extend it by showing that, in voluntary lifestyle sport settings, perceived social rewards such as trust and acceptance may be as important as emotional experiences for identity formation (Giffin et al., 2024). Recent longitudinal evidence similarly indicates that social identification with sport teams predicts athlete wellbeing over time (Graupensperger et al., 2020). The present findings also align with research on psychological belonging, which emphasizes that group memberships become identity-defining when they satisfy needs for connection and esteem (Cruwys et al., 2014; Allen et al., 2022; Hu and Cheung, 2024), and with evidence that social identity shapes positive developmental outcomes in sport contexts (Lee et al., 2023). In lifestyle sport communities, where participation is voluntary and non-contractual, these relational mechanisms may be especially consequential for retention, and social identity has been linked to reduced dropout in sport (Murray and Sabiston, 2022; Wheaton, 2013; Wheaton and Thorpe, 2022).

This finding also reflects the unique characteristics of Ultimate Frisbee clubs. The system of self-officiating, where players act as their own referees to resolve disputes, places trust at the core of every interaction. The mixed-gender format creates diverse opportunities for social connection. Furthermore, the ritual of the post-game spirit circle provides a structured space to affirm social bonds and mutual respect.

## 6 Conclusion

This study elucidates the formation mechanism of group identity in voluntary, non-contractual sports organizations by proposing and testing a dual-pathway model. Integrating Interaction Ritual Chain Theory and Social Exchange Theory, the findings suggest that group identity within Ultimate Frisbee clubs may be shaped jointly by an affective pathway (emotional energy) and a rational pathway (relational benefits). Theoretically, the study extends group identity research from professional spectator sport into active lifestyle sport communities and points to reciprocal social evaluations, particularly trust and acceptance, as a meaningful pathway to identification in settings without formal incentives. Practically, club managers may consider designing ritualized settings that foster co-presence, mutual focus, and emotional entrainment, while also supporting psychological safety, trust, and social acceptance to support long-term member retention.

Several limitations qualify these conclusions. The sample was drawn primarily from first-tier Chinese cities, which limits geographic and cultural generalizability. All quantitative variables were measured by self-report at a single time point; although the Harman single-factor test suggested that common method bias was not a major concern (32.46% of total variance in the first factor), self-report and cross-sectional designs preclude causal inference and cannot fully rule out common method variance. The scales were developed for this investigation and have not yet been independently validated in a separate sample. The qualitative phase was partially theory-informed, and the study was not preregistered; both features are acknowledged for transparency. Finally, emotional energy and relational benefits may influence one another over time, and the relative strength of the two pathways should be interpreted as descriptive rather than causal.

Future research should employ longitudinal and experience-sampling designs to examine identity trajectories and reciprocal dynamics, test the measurement model in other lifestyle sports and cultural contexts, and use multilevel models that account for nesting of members within clubs. Pre-registered hypotheses and analysis plans would further strengthen confidence in the robustness of the dual-pathway model.

## Data Availability

The raw data supporting the conclusions of this article will be made available by the authors, without undue reservation.
