# Supplementary material for: From interaction to identification: a mixed methods study on the formation mechanism of group identity among Ultimate Frisbee club members
Source: Front Psychol. 2026 Sep 28;17:1878237. doi: 10.3389/fpsyg.2026.1878237 (PMC13656089; doi:10.3389/fpsyg.2026.1878237)
Supplement: Supplementary file 1 [file Table_1.docx]

**Supplementary Material**

Manuscript 1878237: From Interaction to Identification: A Mixed Methods Study on the Formation Mechanism of Group Identity Among Ultimate Frisbee Club Members

This file contains Appendix A (participant demographics), Appendix B (open-coding example), and Appendix C (final measurement scale), which are referenced in the manuscript.

# Appendix A. Participant Demographics

Characteristics of the 22 interviewees in Study 1.

| **ID** | **Gender** | **Occupation** | **Playing experience** | **Club role** | **Duration / format** |
| --- | --- | --- | --- | --- | --- |
| 1 | Female | Employed | 2 years | Member | 45 min / offline |
| 2 | Female | Student | Less than 1 year | Member | 45 min / offline |
| 3 | Female | Student | 1 year | Captain | 45 min / offline |
| 4 | Male | Employed | 3 years | Captain | 60 min / online |
| 5 | Male | Employed | 14 years | Manager | 60 min / online |
| 6 | Female | Student | 2 years | Captain | 45 min / online |
| 7 | Female | Employed | 2 years | Member | 45 min / online |
| 8 | Female | Employed | 2 years | Member | 45 min / online |
| 9 | Female | Student | 3 years | Member | 45 min / online |
| 10 | Female | Employed | 4 years | Manager | 60 min / online |
| 11 | Female | Employed | 4 years | Member | 45 min / offline |
| 12 | Female | Employed | 3 years | Manager | 45 min / online |
| 13 | Female | Employed | 2 years | Member | 60 min / online |
| 14 | Female | Employed | 2 years | Member | 60 min / online |
| 15 | Male | Employed | 5 years | Captain | 60 min / online |
| 16 | Male | Employed | 6 years | Member | 45 min / offline |
| 17 | Male | Student | 2 years | Member | 45 min / offline |
| 18 | Male | Student | Less than 1 year | Member | 45 min / offline |
| 19 | Male | Employed | 3 years | Member | 45 min / offline |
| 20 | Male | Employed | 3 years | Captain | 60 min / online |
| 21 | Male | Employed | 3 years | Manager | 45 min / offline |
| 22 | Male | Employed | Less than 1 year | Member | 45 min / offline |

# Appendix B. Open-Coding Example

Representative examples of initial-concept extraction from interview data. Due to space constraints, only part of the coding process is shown; 90 initial concepts were identified in total.

| **Original quote (Chinese)** | **English gloss** | **Initial concept** |
| --- | --- | --- |
| 现在每个队伍都有自己的队服，不然就让人觉得很不正规。（7-4） | Every team has its own uniform; otherwise it feels unprofessional. | Wearing team uniform |
| 每次比赛前，大家都会肩膀搭肩膀，一起喊那个口号，振奋人心。（10-19） | Before each game, everyone links arms and shouts the team cheer together. | Shouting the team cheer |
| 哪怕我在比赛上某个盘没丢好或者失误了，队员也不会凶我。（1-3） | Even when I made a mistake on the field, teammates did not blame me harshly. | Friendly communication |
| 我第一次参加社会俱乐部，因为我的好朋友在这里，可以一起训练打比赛，会比较有安全感吧。（17-3） | I joined a social club because my good friends were here, so training and competing felt safer. | Sense of security |
| 在这里找到了好多同样喜欢户外、喜欢运动的朋友，特别聊得来。（13-2） | I found many friends who also enjoy outdoor activities and sports. | Building friendships |
| 进攻的时候，队友都会帮忙喊“inside”或者“up”，会觉得特别有用。（13-8） | Teammates call “inside” or “up” during offense, which feels very useful. | Sideline support |
| 决赛Uni绝杀后，你冲向我然后抱住我的那个瞬间，我永远都忘不了。（19-17） | I will never forget the moment you ran to hug me after the championship Uni score. | Uni game-winning moment |
| 有一个队友在比赛的时候，脚踝骨折了，好几个队友都去医院探望他。（7-15） | A teammate broke an ankle during a game, and several teammates visited him in hospital. | Caring |
| 在这里，我可以放心大胆地做自己。（4-17） | Here I can be myself without hesitation. | Being oneself |
| 我敢大胆地长传，是因为我知道我的队友一定能把盘接到。（13-7） | I dare to make long passes because I know my teammates will catch the disc. | Trust |
| 跟他们的关系早就超过了队友，感觉更像是家人一样。（10-9） | Our relationship goes beyond teammates; they feel like family. | Teammates like family |

# Appendix C. Final Measurement Scale

Final 31-item measurement scale used in the formal survey. All items were rated on a 5-point Likert scale (1 = strongly disagree, 5 = strongly agree).

| **Construct** | **Dimension** | **Code** | **Item** |
| --- | --- | --- | --- |
| Interaction Ritual | Co-presence | I1 | During competitions, I travel to the venue with my teammates. |
| Interaction Ritual | Co-presence | I2 | I regularly participate in club training. |
| Interaction Ritual | Co-presence | I3 | I persist in training even in bad weather. |
| Interaction Ritual | Group Boundaries | I4 | I understand the club’s tactical terminology. |
| Interaction Ritual | Group Boundaries | I5 | I participate in the club’s tryouts. |
| Interaction Ritual | Group Boundaries | I6 | The club training I attend is not open to the public. |
| Interaction Ritual | Mutual Focus | I7 | I respond when a teammate makes a good play. |
| Interaction Ritual | Mutual Focus | I8 | I listen attentively when the coach explains tactics. |
| Interaction Ritual | Mutual Focus | I9 | When making a cut, I can sense my teammates’ gaze. |
| Interaction Ritual | Emotional Entrainment | I10 | I resonate with my teammates during the post-game circle. |
| Interaction Ritual | Emotional Entrainment | I11 | My teammates’ training status affects me. |
| Interaction Ritual | Emotional Entrainment | I12 | During games, I stay in sync with the team’s rhythm. |
| Emotional Energy | - | EE1 | The post-game circle energizes me. |
| Emotional Energy | - | EE2 | Participating in club activities boosts my self-confidence. |
| Emotional Energy | - | EE3 | Participating in club activities gives me vitality. |
| Emotional Energy | - | EE4 | I look forward to the club’s next training session. |
| Relational Benefits | Trust Benefits | R1 | Teammates trust me enough to pass me the disc during crucial points. |
| Relational Benefits | Trust Benefits | R2 | I can feel my teammates’ trust in me. |
| Relational Benefits | Trust Benefits | R3 | I have good chemistry with my teammates. |
| Relational Benefits | Social Benefits | R4 | I have made friends within the club. |
| Relational Benefits | Social Benefits | R5 | Teammates actively communicate with me. |
| Relational Benefits | Social Benefits | R6 | Teammates care about my well-being. |
| Group Identity | Cognitive Identity | G1 | Whether winning or losing, I am a member of the club. |
| Group Identity | Cognitive Identity | G2 | When introducing myself, I say “our team” rather than “their team.” |
| Group Identity | Cognitive Identity | G3 | The club is a part of my life. |
| Group Identity | Affective Identity | G4 | The club gives me a sense of belonging, like “home.” |
| Group Identity | Affective Identity | G5 | I am proud to be a member of this club. |
| Group Identity | Affective Identity | G6 | Even when not training, I still think about this club. |
| Group Identity | Evaluative Identity | G7 | I believe my club is better than other clubs. |
| Group Identity | Evaluative Identity | G8 | My view of the club is positive. |
| Group Identity | Evaluative Identity | G9 | I feel offended when outsiders criticize the club. |
